# A novel copper-based nanosystem for augmented breast cancer photothermal and chemodynamic therapy

**DOI:** 10.1039/d5ra08100a

**Published:** 2026-01-08

**Authors:** Mohammad Seyedhamzeh, Farzaneh Falahi, Ali Kalantari-Hesari, Kimia Esmaeilzadeh, Laleh Salarilak, Hadi Bagheri, Shayesteh Bochani, Satar Yousefiasl, Helia Behrouzfar, Mohammad Esmaeil Akbari, Aziz Maleki

**Affiliations:** a Zanjan Pharmaceutical Nanotechnology Research Center (ZPNRC), Zanjan University of Medical Sciences Zanjan 45139-56184 Iran maleki@zums.ac.ir; b Department of Medical Biotechnology, School of Medicine, Zanjan University of Medical Science Zanjan 45139-56111 Iran; c Department of Basic Sciences, Faculty of Veterinary Medicine, Bu-Ali Sina University Iran; d Dental Research Center, Dentistry Research Institute, Tehran University of Medical Sciences Tehran 1417614411 Iran; e Faculty of Veterinary Medicine, Science and Research Branch of Islamic Azad University Tehran Iran; f Cancer Research Center, Shahid Beheshti University of Medical Sciences Tehran Iran; g Department of Pharmaceutical Nanotechnology, School of Pharmacy, Zanjan University of Medical Science Zanjan 45139-56111 Iran

## Abstract

Among the most common cancers, metastasis and recurrence are the major causes of mortality in breast cancer. Current treatments, such as surgery and chemotherapy, have so far been inadequate in managing metastatic disease. This paper focuses on the potential of multi-functional copper-cysteine nanoparticles (Cu-Cys NPs) as a platform that incorporates PTT and CDT against this challenge. The dl- and l-Cu-Cys NPs were constructed through a straightforward coordination reaction between Cu^2+^ and cysteine ligands (dl and l), followed by detailed characterizations regarding their photothermal conversion efficiency and reactive oxygen species (ROS) generation capability. *In vitro* cytotoxicity and apoptosis of the 4T1 breast cancer cells treated with the prepared NPs were studied through MTT and apoptosis assays, respectively. Tumor growth, metastasis, and systemic toxicity *in vivo* were studied in intratumoral administrations in 4T1 tumor-bearing mice. The combined PTT and CDT not only remarkably ablated the primary tumor but also inhibited breast cancer metastasis with high efficiency. This work thus demonstrates the potential of combining PTT and CDT using a single nanosystem for treatment of metastatic tumors.

## Abstract 2

This work develops and characterizes copper-cysteine nanoparticles as a multifunctional nanoplatform combining photothermal therapy and chemodynamic therapy to ablates primary tumors and inhibit metastatic breast cancer cells in tumor-bearing mice.

## Graphical abstract

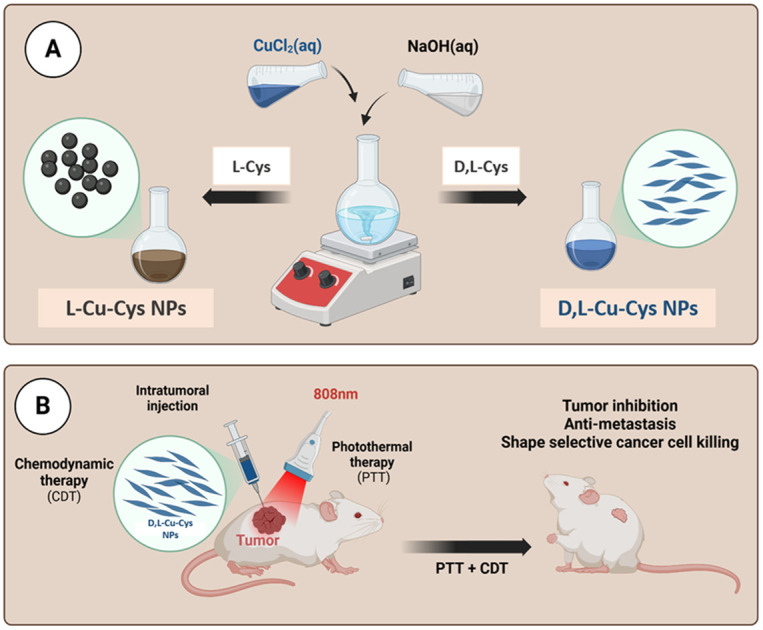

## 1. Introduction

Breast cancer has globally been the leading diagnosed cancer in females.^1^ At present, surgery and chemotherapy are common methods to treat primary breast cancer.^2^ The main limitation of the methods is cancer metastasis and recurrence, contributing to the majority of cancer-related deaths worldwide.^3^

Photothermal therapy (PTT) has attracted tremendous attention in the past decades due to its non-invasiveness, high efficiency, and spatio-temporal selectivity. Using this approach near-infrared (NIR) light is absorbed by a photothermal agent, producing heat upon irradiation by the light, thus leading to elevated local tumor temperature and, ultimately causing irreversible tumor cell death. In addition, recent studies have also witnessed that PTT can boost immunological responses to eliminate the remaining cancer cells, thus preventing further metastasis.^4–6^ PTT may also be combined with other therapeutic approaches such as chemodynamic therapy, photodynamic therapy, gene therapy, and chemotherapy to realize improved therapeutic outcomes.^7,8^ Chemodynamic therapy (CDT) has emerged as a novel therapeutic modality utilizing Fenton or Fenton-like reactions to destroy cancer cells *via* conversion of endogenous H_2_O_2_ in the tumor microenvironment (TME) to highly cytotoxic hydroxyl radicals (˙OH).^9,10^

In 2023, an *in situ* activatble nitrobenzene-cysteine-copper(ii) nano-complexes was developed for programmed photodynamic cancer therapy. The nanosystem showed outstanding potential such as chemodynamic and photodynamic therapeutic performance as well as anticancer effect by anti-angiogenesis in TME under light irradiation (532 nm).^11^ Combining PTT and radiotherapy using a single nanoagent CuS/[^131^I]I was used for imaging-guided treatment of metastatic tumors.^7^ It was demonstrated that the PEG-functionalized NPs could migrate to and remain in their nearby sentinel lymph nodes after being injected into primary solid tumors. The combined PTT and radiotherapy significantly prolonged animal survival and inhibited cancer metastasis remarkably, highlighting importance of the combination therapy in tumor elimination and overcoming cancer metastasis.

It has been reported that Cu-catalyzed Fenton-like reaction could occur in acidic TME with much higher catalytic efficiency (∼160 tfimes than that of Fe^2+^-catalyzed Fenton-like reaction).^12,13^ Therefore, copper-based nanosystems with high catalytic performance and specificity are highly desirable to be developed as a chemodynamically active nanostructures for robust ROS generation.^14–17^ Besides, Cu-based NPs have a strong absorption at the NIR region, thus allowing tumor PTT.^4,18^

**Fig. 1:**
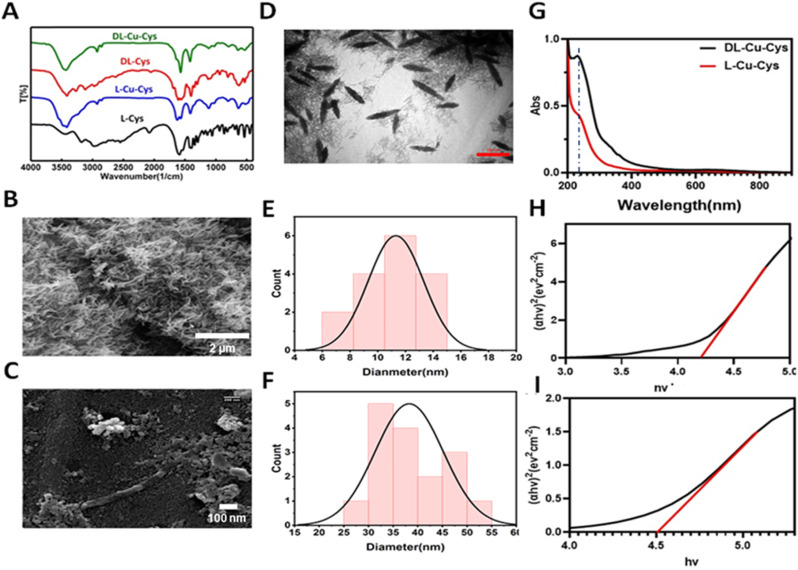
(A) FTIR spectrum of l-cysteine (black), l-Cu-Cys (blue), dl-cysteine (red), and dl-Cu-Cys (green). SEM image of dl-Cu-Cys (B), and l-Cu-Cys (C) TEM image (D) and (E and F) width and length distribution of dl-Cu-Cys. (G) UV-vis spectrum of l-Cu-Cys (red) and dl-Cu-Cys (black). Tauc plot of dl-Cu-Cys (H) and l-Cu-Cys (I).

**Scheme 1:**
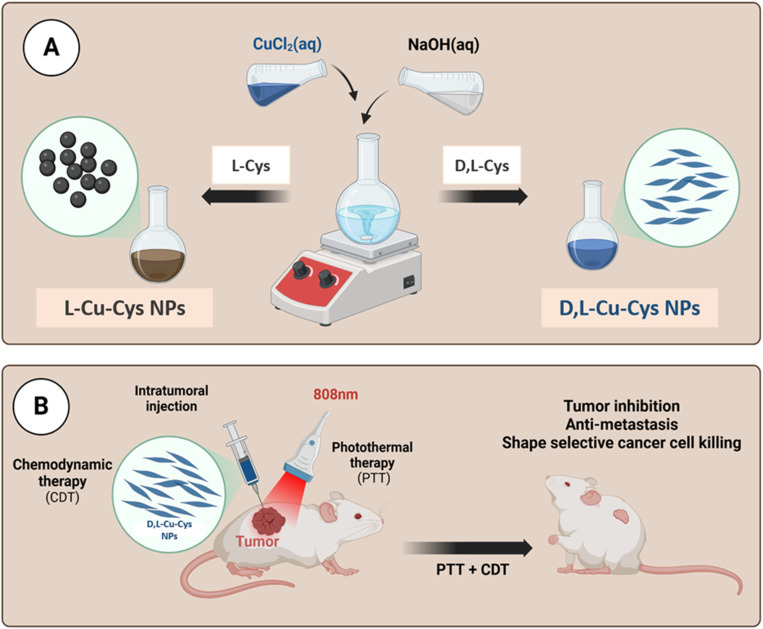
Schematic representation of the fabrication and therapeutic application of Cu-Cys NPs (created by Biorender).

Herein, a novel chemodynamically and photothermally active copper-containing nanosystem was developed as a proof of concept to effectively enhance photo-induced Fenton-like for tumor therapy. As shown in Fig. 1A, copper-cysteine NPs (Cu-Cys NPs) were fabricated *via* a facile coordination process between Cu^2+^ and the l-cysteine and dl-cysteine, named as l-Cu-Cys and dl-Cu-Cys, respectively. Different morphologies, *i.e.*, spherical and rice shapes, were achieved when the two different ligands were used as ligands in the preparation process of the NPs. Compared to l-Cu-Cys NPs, cancer cell killing significantly increased when dl-Cu-Cys NPs were used. Our *in vitro* achievements revealed that a large amount of ROS was generated *via* copper-catalyzed Fenton-like reactions. In 4T1 tumor-bearing mouse model, as a result of produced hyperthermia, the dl-Cu-Cys NPs were able to remarkably reduce the tumor growth and breast cancer-to-lung metastasis. As a result, our approach to boosting ROS generation combined with PTT shows that dl-Cu-Cys NPs could be a promising clinical nanoplatform for efficient tumor treatment (Scheme 1).

## 2. Materials and methods

### 2.1. Materials

Sodium hydroxide (NaOH), d-l-cysteine, ethanol, methyl blue (MB), 3-(4, 5-dimethylthiazol- 2 yl)-2, 5-diphenyltetrazolium bromide (MTT), 3,3′,5,5′-tetramethylbenzidine (TMB), 1,3-diphenylisobenzofuran (DPBF), 2′, 7′-dichlorofluorescein diacetate) (DCFDA), and CuCl_2_·2H_2_O were purchased from Merck company. The chemicals were of analytical grade and used without further purification.

### 2.2. Preparation of Cu-Cys NPs

For the construction of Cu-Cys NPs, a 17 mg solution of CuCl_2_ was dissolved in deionized water. In a separate vial, 24 mg of dl or l-cysteine was dissolved in 1 mL of aqueous NaOH (0.6 M). The cysteine solution was slowly added to the CuCl_2_ (aq) and mixed for 3 minutes to create Cu-Cys NPs. Subsequently, the reaction mixture underwent centrifugation at a speed of 10 000 rpm and then washed using water and ethanol (1 : 1). The NPs were ultimately dispersed in water and stored in a refrigerator for further investigations.^19^ The same procedure was used to prepare l-Cu-Cys NPs.

### 2.3. Characterization of Cu-Cys-NPs

The morphology, particle size, and elemental content of Cu-Cys NPs were analyzed by Transmission Electron Microscopy (TEM) (Philips EM208S, Netherlands) and Scanning Electron Microscopy (Quanta 250 FEG, USA). The Zeta potential of the particles was determined using Dynamic Light Scattering (DLS) with the Zetasizer Nano ZS instrument from Malvern Panalytical Ltd, located in Malvern, UK. The surface functional groups of the NPs (NPs) were analyzed using Fourier Transform Infrared Spectroscopy (FTIR) in the wavelength range of 400–4000 cm^−1^ (FTIR, Bruker, Tensor 27, Germany). The UV-vis-NIR analysis of NPs was studied by a UV-vis spectrophotometer (Bruker IFS, 66/*vs.*).

### 2.4. Reactive oxygen species detection

The production of hydroxyl radicals (˙OH) was evaluated using the degradation of methylene blue (MB) by the hydroxyl radicals. This was accomplished by measuring the absorbance at 665 nm. To this end, dl- and l Cu-Cys NPs (100 µg mL^−1^) were combined with H_2_O_2_ (200 µL, 10^−3^M) and MB solution (1 mL, 0.001 M), and were subjected to 808 nm light exposure (1 or 1.5 W cm^−2^) for 8 min. Subsequently, the absorbance of the solutions was measured at a wavelength of 652 nm. The experiments were conducted in duplicate.^20^ In addition, to evaluate ROS, a solution containing Cu-Cys NPs (100 µg mL^−1^) and DPBF (40 µg mL^−1^) was prepared in 3.0 mL of PBS (0.1 M, pH 7.4). Next, the generation of the ROSs by the NPs was measured by monitoring the absorbance changes of DPBF at 416 nm after NIR irradiation (808 nm, 1 W cm^−2^). An experiment without NIR light exposure was considerd as a control.^21^

### 2.5. *In vitro* degradation of the Cu-Cys-NPs

300 µL of the synthetic NPs (1 mg mL^−1^) was added to 3 mL of 1 mM H_2_O_2_ (35% w/v) solution and then incubated at 37 °C for 30 minutes. The *in vitro* degradation was then assessed by SEM (SEM, Quanta 250 FEG, USA) and UV-Vis spectroscopy (Bruker IFS, 66/*vs.*).^22^

### 2.6. Photothermal performance measurement

The photothermal activity of Cu-Cys NPs (500 µL aqueous solution) with different concentrations (200 µg mL^−1^ and 400 µg mL^−1^) was investigated under 808 nm NIR laser (3L-IR, Hamerz Rad) at power densities of 1000 and 1500 mW for 8 min. The temperature of the samples was recorded using an infrared camera (TiS55, Fluke, USA). The photothermal stability of Cu-Cys NPs (400 µg mL^−1^) was explored by four cycles of laser on-off.^19^ In addition, the photothermal conversion efficiency (*η*) of the nanostructures was measured according to the previous method.^23,24^

### 2.7. Cell viability assay

The 4T1 (metastatic adherent epithelial murine breast cancer cells) were purchased from Royan Institute of Iran and were cultured in Dulbecco's Modified Eagle Medium (DMEM) supplemented with 10% fetal bovine serum (FBS) and 1% penicillin-streptomycin at 37 °C in a 5% CO_2_ atmosphere. The cytotoxic effects of dl-Cu-Cy NPs were evaluated using the 3-(4, 5-dimethylthiazol-2-yl)-2, 5-diphenyltetrazolium bromide (MTT) assay. Briefly, 0.6 ×10^4^ cells per well were placed in each well of a 96-well plate. Upon reaching 90% confluence, the media were extracted and the cells were subjected to escalating concentrations of dl-Cu-Cy NPs (0–40 µg mL^−1^). After 4 h, one group was exposed to 808 nm laser irradiation (1 W cm^−2^, 8 min), while another group was left unexposed. Next, both groups were incubated at 37 °C for 48 h. The group receiving no treatment was considered as the control group. Subsequently, the media were aspirated and then a diluted solution of MTT (5 mg mL^−1^) was added, followed by another incubation for 4 h at 37 °C to allow the formation of formazan crystals. Afterward, the produced formazan crystals were solved in 150 µL of dimethyl sulfoxide (DMSO) and the absorbance at 570 nm was measured using a microplate reader (BioTek). The cell viability percentage was determined using the following formula:
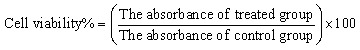


### 2.8. Cell apoptosis assays

Cell apoptosis assays was evaluated by Annexin V-FITC and propidium iodide staining of the 4T1 cell line and flow cytometry method.^16^ In each 6-well plate, 30 × 10^4^ cells were seeded, and the following day, the media were replaced with fresh media containing 20 µg mL^−1^ of dl-Cu-Cy NPs. The control group remained untreated and was only provided with fresh media. Each experimental group was replicated three times. After a 5 h incubation period, one treated group was exposed to irradiation (808 nm, 1 W cm^−2^, 8 min), while the remaining groups were left untreated. After 48 h, the media were removed, and cells from each group were washed with PBS, trypsinized, and then incubated for 15 minutes in 1000 µL of binding buffer. Then 6 µl of Annexin V-FITC and 3 µl of propidium iodide were added at room temperature in the darkness. Subsequently, the cells were subjected to flow cytometry analysis using a flow cytometer (BD Accuri C6 plus). The FlowJo 10 software was utilized for data analysis.

### 2.9. Live/dead fluorescent imaging

The cells were treated and irradiated according to the previously established protocol for the apoptosis assay.^25^ After 48 h post-irradiation, the media were aspirated, and the cells were washed with PBS before being incubated in 1 mL PBS containing 1 µL calcein-AM and 2 µL PI at 37 °C. Afterward, the cells were washed twice with PBS, and images were captured using fluorescent microscopy (Olympus) with red (indicating dead cells) and green (indicating live cells) filters. The images were then analyzed by ImageJ software and the live/dead ratio in each groups was quantified.

### 2.10. *In vitro* cell migration assay

For the scratch assay, 15 × 10^4^ cells per well were cultured in 12-well plates and allowed to form a monolayer. Subsequently, the culture media were replaced with fresh media in the control group, and fresh media containing dl-Cu-Cy NPs at a concentration of 20 µg mL^−1^ in two other groups.^26^ After 5 hours, one of the treated groups was subjected to irradiation (808 nm, 1 W cm^−2^, 5 min), while the others were not irradiated. A 200 µL plastic pipette tip was applied to scratch a vertical wound, and cellular debris was removed by washing the cells with PBS. The wells were then refilled with serum-free culture medium. Next, the cells were promptly imaged, and photography was continued every 24 h until the gap in the control group was filled. The area of gap filling was quantified using ImageJ software, and the degree of migration inhibition was computed using the subsequent formula.^27^
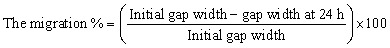


### 2.11. Intracellular ROS generation

The detection of ROS was performed with reactive oxygen species assay kit (Kiazist, Iran). In brief, 25 × 10^4^ cells were seeded into each well of a 24-well plate. Before treatment, 400 µL DCFDA reagent (20 µM) was added to each well and incubated in the dark for 40 min. After 1 h, the cells were treated with 20 µg mL^−1^dl-Cu-Cy NPs and one group was left untreated as a control group and incubated at 37 °C. After 5 h of incubation, one group (dl-Cu-Cy (+NIR)) was irradiated (808 nm, 1 W cm^−2^, 8 min) and the other groups remained non-irradiated. Afterward, the media was removed in all groups and the cells were washed 3 times with PBS and ROS buffer. Then ROS generation in each group was observed by fluorescence microscopy (Olympus).^28^

### 2.12. Animal studies

Mice were housed at the Animal Center of Zanjan University of Medical Sciences (Iran). Guidelines stated in the Guide for the Care and Use of Laboratory Animals by the US National Institutes of Medicine were adhered to. The study protocol was approved by the Cancer Research Centre, Shahid Beheshti University of Medical Sciences, Tehran, Iran (IR.SBMU.CRC.REC.1400.028).

### 2.13. *In vivo* hyperthermia-enhanced nanozyme catalytic treatment effect

The antitumor effect of dl-Cu-Cys NPs was assessed in a subcutaneous 4T1 tumor model. When the tumor volume reached ∼100 mm^3^, mice were randomly divided into 3 groups, PBS, dl-Cu-Cys (+NIR), and dl-Cu-Cys (-NIR) (*n* = 5). The mice were intratumorally administered with dl-Cu-Cys (2 mg kg^−1^, 100 µL) on days 1 and 3. The dl-Cu-Cys (+NIR) group was illuminated by a NIR light (808 nm, 1 W cm^−2^) for 5 min. The body weight and tumor volume were recorded every 2 days. The mice were sacrificed on the 14th day, and tumors, the lung, and the liver of the mice were collected for hematoxylin and eosin (H & E) staining. The tumor volume (*V*) was calculated as following furmula:*V* = width^2^ × length/2.

### 2.14. Histopathological analysis

On day 14, the lung, spleen, and tumor tissues were collected and fixed with 10% neutral buffered formalin for hematoxylin and eosin (H & E) staining. The organs were dehydrated clarification with xylene, paraffin-embedded, sectioned using a microtome, and stained using the H & E method. The histopathological study was examined by a microscope, Dino-Lite camera, and Dino-capture software (V.2).

### 2.15. Statistical analysis

Data analyses were performed with Origin Pro 9.1 and GraphPad Prism 7.0. The experimental results were presented as mean ± SD (standard deviation). *T*-test and one-way ANOVA statistical analysis were used to calculate the significant difference between different groups. *p < 0.05, **p < 0.01, ***p < 0.001, and ****p < 0.0001 were considered to be statistically significant.

## 3. Results and discussion

### 3.1. Fabrication and characterization of dl and l-Cu-Cys-NPs

FTIR spectra of dl and l-Cu-Cys NPs are presented in Fig. 1A. All samples exhibited a broad peak at around 3410 cm^−1^, which is assigned to the N–H stretching vibrations of the amine group in cysteine. A peak at 2500–2600 cm^−1^ can be related to –SH group of dl-Cys and l-Cys.^19^ Intensity of the peak disappeared when the dl-Cu-Cys and l-Cu-Cys NPs were formed, confirming the involvement of a thiol group through a Cu–S interaction in the NPs. Interestingly, as shown in Fig. 1B–D, when dl-Cys and l-Cys were used, the resulting Cu-Cys NPs exhibited rice and spherical morphologies respectively. This was confirmed by TEM and SEM images, indicating the impact of the stereochemistry of cysteine ligands on the shape of the nanostructures.^29,30^ After incubation H_2_O_2_ with dl-Cu-Cys, SEM analysis revealed that the morphology of the nanostructures changed completely (Fig. S1). This was good document confirming the TME responsiveness of the nanostructures, thus acting as a chemodynamically active particle.^12^ Such activity, will be further proved using following experiments. TEM analysis of dl-Cu-Cys indicated a rice-like structure with an average width of 10.56 ± 3.44 nm and a length of 38.25 ± 6.99 nm (Fig. 1D–F). In addition, the band gaps of the Cu-Cys NPs were determined by the Kubelka–Munk method.^31^ According to Fig. 1H and I, the determined band gaps of dl-Cu-Cys NPs and l-Cu-Cys NPs are 4.21 eV and 4.51 eV, respectively. These results prove that the dl-Cu-Cys NPs exhibited a narrower bandgap than the l-Cu-Cys NPs. It has been reported that, a narrower band gap endowed with a nanosystem with efficient light absorption capacity, thus generating electron–hole pairs to drive the process of photocatalytic reactions.^32,33^

According to the UV-vis spectra, the Cu NPs characteristic absorbance peak (∼240 nm) were observed for both dl-Cu-Cys and l-Cu-Cys NPs (Fig. 1G).^34^ A slight red shit was observed in the l-Cu-Cys NPs, showing a different interaction between l-cysteine and copper centers. In addition, the absorption peak centered at 240 nm completely disappeared in the UV-vis spectra of dl-Cu-Cys after incubation of the nanostructures with H_2_O_2_ (Fig. S2). These results confirmed that the nanosystem was degraded by H_2_O_2_ which is overexpressed in the tumor microenvironment (Fig. S2).^35^

**Fig. 2:**
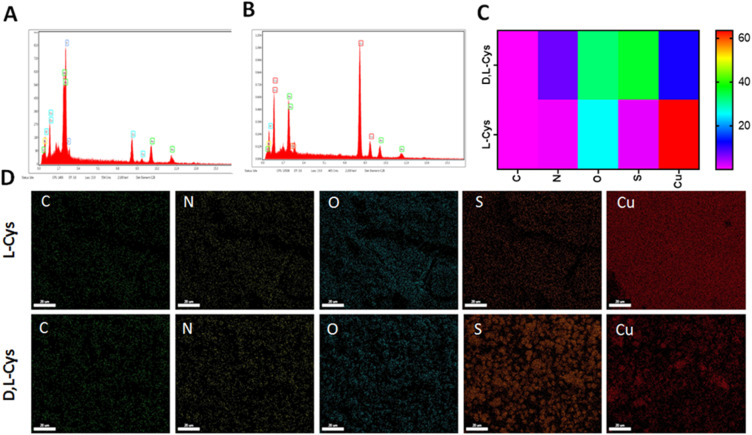
EDX analysis of l-Cu-Cys (A) dl-Cu-Cys (B) and its related Heat map Plot (C), and (D) elemental map analysis of l-Cu-Cys and dl-Cu-Cys NPs.

The energy dispersive X-ray (EDX) analysis provides evidence for the co-existence of Cu and S atoms in the Cu-Cys NPs (Fig. 2A–C). The EDX analysis confirmed the successful incorporation of copper. For the dl-Cu-Cys and l-Cu-Cys nanosystems, the copper content was found to be 64.74 wt% and 15.01 wt% respectively. The uniform distribution of Cu, S, O, C, and N in the elemental mapping images illustrated its structural integrity and successful formation of dl and l-Cu-Cys NPs with high dispersity (Fig. 2D).

### 3.2. *In vitro* photothermal and chemodynamic assessments

**Fig. 3:**
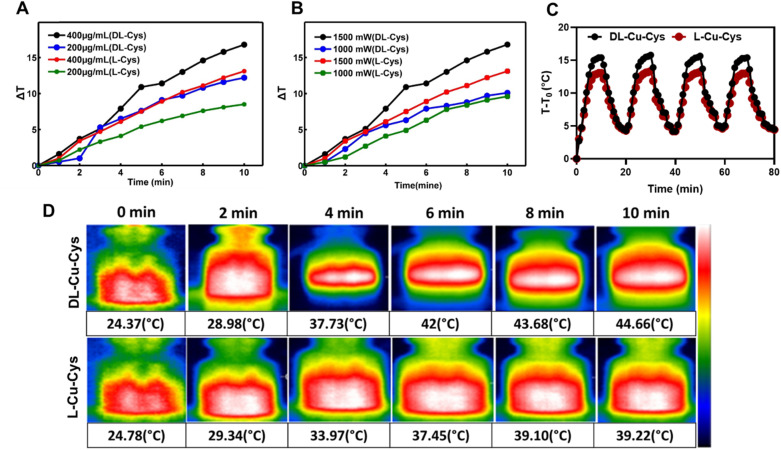
Temperature curves of dl-Cu-Cys and l-Cu-Cys NPs at (A) different concentration (200 and 400 µg mL^−1^, under 808 nm laser irradiation and power density), (B) different power density (1.0 and 1.5 W cm^−2^), (C) temperature changes of dl-Cu-Cys and l-Cu-Cys NPs in 4 laser on/off cycles of irradiations, (D) the NIR thermal images of the dl-Cu-Cys and l-Cu-Cys for 8 min (808 nm, 1.5 W cm^−2^).

In order to assess and compare the photothermal efficacy of Cu-Cys NPs, various concentrations (200 µg mL^−1^ and 400 µg mL^−1^) of the prepared NPs and power densities (1000–1500 mW) were used at a wavelength of 808 nm for a duration of 10 minutes (Fig. 3A and B). The resulting temperature changes were monitored in real-time. It is evident that when the concentration and power increase, there is a more significant rise in temperature, particularly for dl-Cu-Cys NPs compared to l-Cu-Cys NPs. This behavior can be attributed to the higher photothermal conversion efficiency (PTCE) of dl-Cu-Cys NPs. Our results showed that the PTCE of dl-Cu-Cys NPs (22.4%) was higher than that of the l-Cu-Cys NPs (14.3%), highlighting the shape dependency of the PTCE (Fig. S3).^36^ This finding is in good agreement with bandgap results of the nanostructures (Fig. 1H and I). Moreover, both NPs still maintained excellent photothermal properties after the on/off laser cycle for 4 times (Fig. 3C). Hence, the NPs as a multifunctional copper-carried havebeen successfully prepared with an appropriate photothermal activity for tumor combination therapy. Fig. 3D shows IR thermal images of the dl-Cu-Cys and l-Cu-Cys NPs under continuous laser irradiation for 10 min, confirming the time dependency of the photothermal behavior. In addition, we didn't observe any temperature increase in water as control group, thereby confirming that the photothermal effect is due to the existence of copper nanostructures (Fig. S3).

**Fig. 4:**
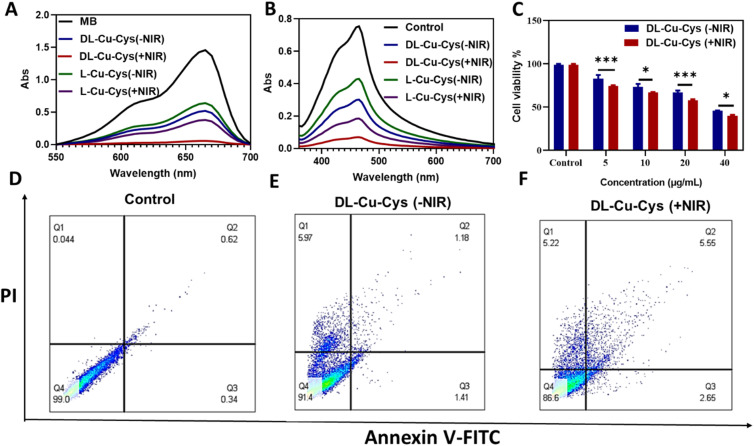
(A and B) UV-vis spectrum curves of MB and DPBF treated with dl-Cu-Cys and l-Cu-Cys NPs in the presence or absence of NIR light, (C) the percent viability of 4T1 cells treated with different concentrations of dl-Cu-Cys NPs after 48 hours in the MTT assay. Data are presented as mean ± SD (*n* = 3). *P < 0.05, and ***P < 0.001, and (D–F) Apoptosis assay for 4T1 cells incubated with 20 µg dl-Cu-Cys NPs with or without NIR (808 nm, 1 W cm^−2^, and 8 min) by flow cytometry.

Previous studies have shown that Cu-Cys NPs exhibited a remarkable catalytic activity through a Fenton-like reaction between Cu^2+^ and H_2_O_2_.^19,37^ As shown in Fig. 4A, the amino acid type influenced peroxidase-like catalytic activities of Cu-Cys NPs, when methylene blue (MB) was used as a molecular probe to detect hydroxyl radicals (˙OH).^38^ Obviously, in comparison with l-Cys, the MB absorbance significantly reduced because of the activity of dl- Cu-Cys NPs. Such activity was more pronounced when NIR light was used, confirming NIR-accelerated chemodynamic therapy (Fig. 4A). ^1^O_2_ generation, as another type of ROS, was assessed by 1,3-diphenylisobenzofuran (DPBF) as an indicator (Fig. 4B).^39^ Compared to ROS detection using MB, the same trend was observed when DPBF was utilized NIRa probe, again implying thermo-responsive property of the ^1^O_2_ production.^40,41^

### 3.3. dl-Cu-Cy NPs could enhance ROS formation and induce cell death and apoptosis in 4T1 cells upon NIR irradiation

The MTT assay was conducted to investigate the cytotoxicity and PTT effect of dl-Cu-Cys NPs on 4T1 cells. As illustrated in Fig. 4C, the cell viability in the presence of the NPs reduced in a dose-dependent manner, and this cytotoxic effect was further enhanced when combined with the NIR light. Furthermore, to investigate the cellular death process after the laser irradiation, apoptosis/necrosis was assessed using Annexin V-FITC/PI staining. As illustrated in Fig. 4D–F, apoptosis and necrosis was observed in the dl-Cu-Cys (−NIR) and dl-Cu-Cys (+NIR) groups when compared with control group. The proportion of apoptotic/necrotic cells were higher in the presence of NIR light. Previous studies show that a temperature of more than 42 °C (considered in PTT) can cause irreversible tissue damage and subsequently trigger cell apoptosis.^42,43^ Recent studies have shown that Cu-based NPs have a high potential for PTT, as these particles have high NIR absorption and can effectively convert NIR light energy into heat.^44,45^

**Fig. 5:**
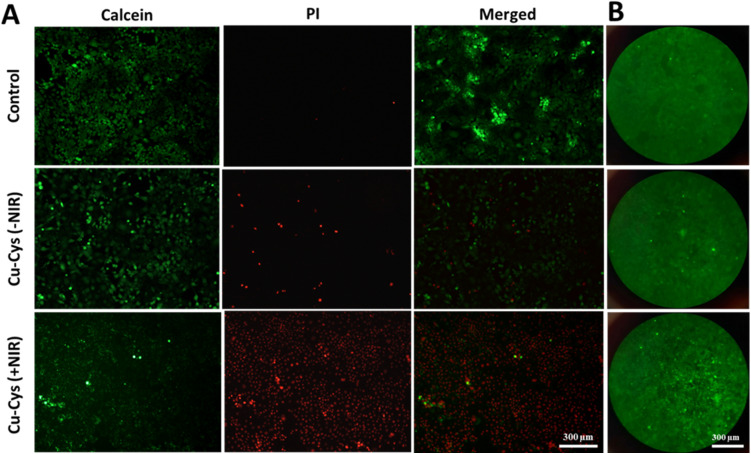
(A) Live/dead fluorescence imaging of 4T1 cells after incubation with 20 µg mL^−1^dl-Cu-Cys NPs with or without NIR (808 nm, 1 W cm^−2^, and 10 min) by calcein-AM/PI staining. Live cells stained with calcein-AM in green color and dead cells stained with PI in red color (magnification; 10X, scale bar; 300 µm) and (B) fluorescence images of intracellular ROS levels of 4T1 cells incubated with 20 µg mL^−1^dl-Cu-Cys NPs with or without NIR (808 nm, 1 W cm^−2^, and 5 min) performed by DCFH-DA staining. (Magnification; 10X, scale bar; 300 µm).

To confirm the photothermal impact caused by dl-Cu-Cys NPs, live/dead staining was performed using 20 µg mL^−1^dl-Cu-Cys NPs (Fig. 5A). In this experiment, live cells were stained in green and dead cells turned red. As depicted in Fig. 5A, the majority of cells in the control group appeared green, in contrast, the number of red cells increased in the presence of dl-Cu-Cys NPs without the laser irradiation. The highest number of dead cells was observed in the cells treated with the dl-Cu-Cys(+NIR) group, revealing the impact of the generated local heat in the killing of 4T1 cells.^46^ The analysis of the live/dead ratio showed that dl-Cu-Cys (+NIR) group showed a decrease compared to the dl-Cu-Cys (−NIR) group and control groups. Also, compared to the control group, there was no significant change in dl-Cu-Cys (−NIR) group (Fig. S4).

To investigate ROS formation in 4T1 cells treated with dl-Cu-Cys NPs with or without NIR irradiation, a ROS assay was performed using DCFDH-DA as probe. As shown in Fig. 5B, the intensity of green fluorescence was not significant in the control group, while the intensity of green fluorescence increased slightly in the cells treated with dl-Cu-Cys (−NIR) group. Compared to the other groups, the highest intensity of green fluorescence was observed in the dl-Cu-Cys (+NIR) group. This result indicated that the Cu-based NPs were able to enhance ROS formation when exposed to irradiation *via* Fenton-like reaction.^47,48^

### 3.4. dl-Cu-Cy NPs reduced the migratory capacity of 4T1 cells individually or when exposed to NIR

**Fig. 6:**
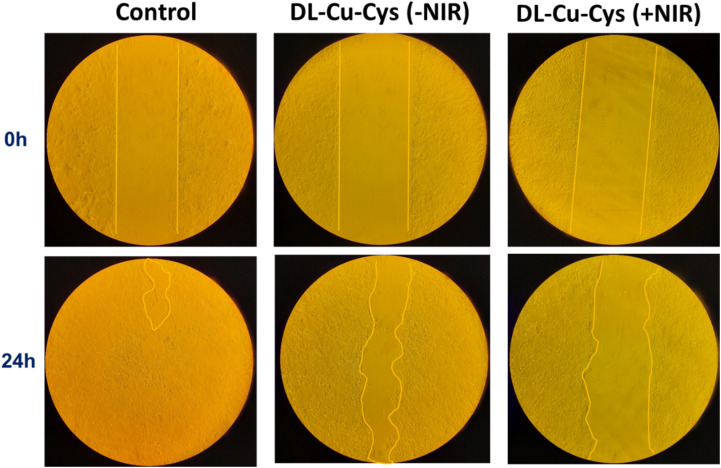
Migration of 4T1 cells treated with 10 µg mL^−1^dl-Cu-Cys NPs with or without NIR (808 nm, 0.85 W cm^−2^, and 5 min) was assessed by scratch assay and photographed during 24 hours.

A scratch assay was performed to assess the impact of dl-Cu-Cys NPs with and without 808 nm laser irradiation on the cellular migration of 4T1 cells.^49^ In this experiment, a concentration of 10 µg mL^−1^ of the NPs and 0.85 W cm^−2^ was utilized, which minimally impacted cell viability. As illustrated in Fig. 6 and S5 the cells in the control group nearly closed the gap after 48 h, whereas the group treated with dl-Cu-Cys (−NIR) exhibited a 35.92% reduction in migration compared to the control. Furthermore, this reduction was further heightened to 63.54% in the group treated with dl-Cu-Cys (+NIR). These results underscored the inhibitory effect of the Cu-based nanostructures on 4T1 cell migration, which was further enhanced in combination with the 808 nm laser illumination.^50^

### 3.5. *In vivo* anticancer assay

**Fig. 7:**
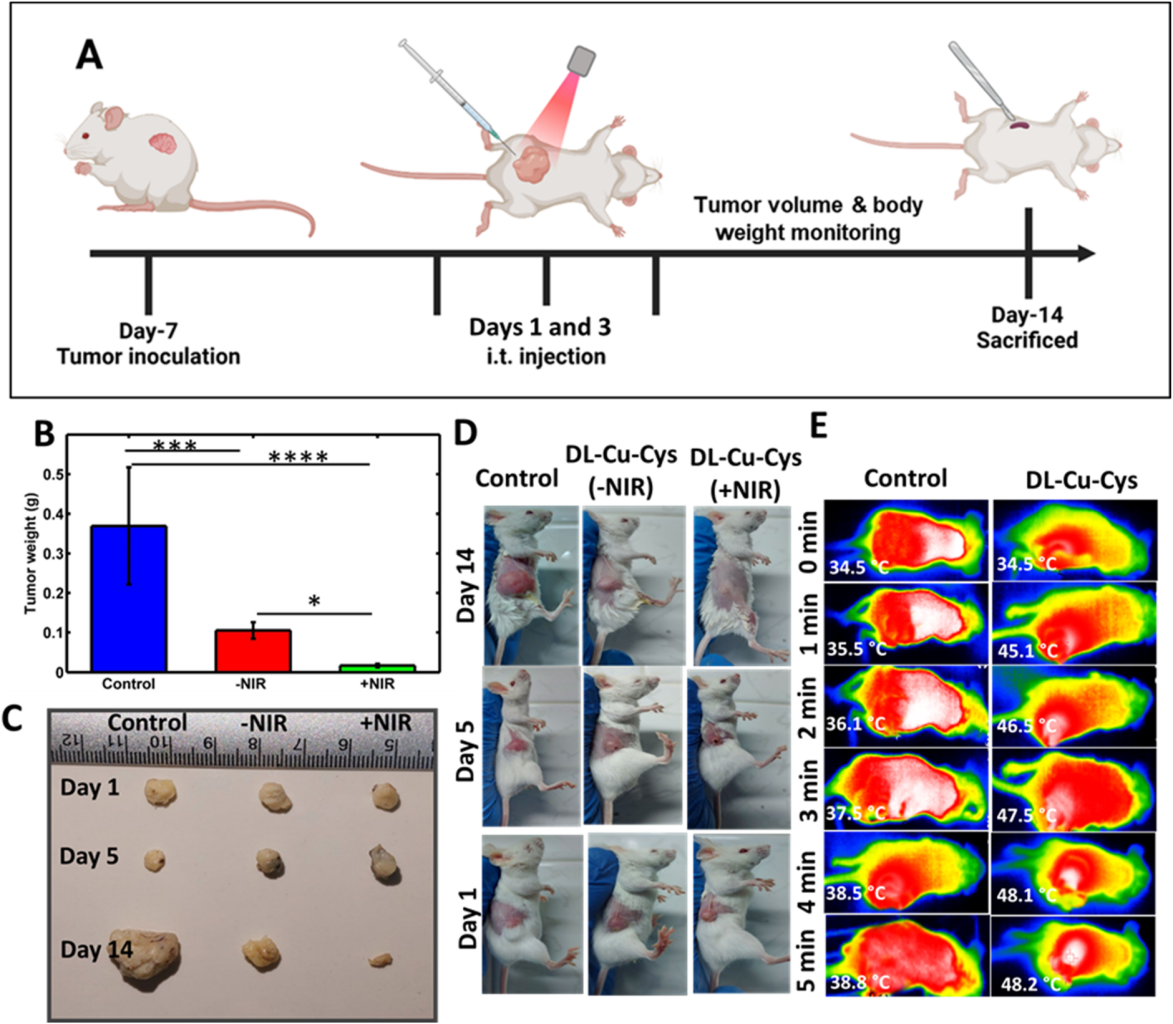
(A) Therapeutic schedule for 4T1 tumor-bearing mice. (B) Monitoring assay for the change of tumor weight over 14 days. The error bar shows mean ± SEM with two-way ANOVA, Tukey's multiple comparisons tests; *p < 0.05,***p < 0.001, and ****p < 0.0001. (C) Photos of the tumors on days 1, 5, and 14. (D) Typical photographs of mice to assess the tumor volume after various treatments, and (E) infrared thermal images of tumor-bearing mice after intratumoral injection of PBS or dl-Cu-Cys NPs.

The *in vitro* and in-cell studies confirmed the efficient tumor cell-killing effect of dl-Cu-Cys NPs. Therefore, we further investigated the therapeutic efficacy of dl-Cu-Cys NPs in the 4T1 subcutaneous tumor model. The mice were intratumorally injected with the NPs and, then irradiated with a laser at 1.0 W cm^− 2^ with a temperature of ∼50 °C during the whole irradiation time. The treatment schedule is shown in Fig. 7A. We observed that within 14 days, the tumor weight increased to approximately 350 mg in mice administered by PBS (Fig. 7B). For the dl- Cu-Cys (−NIR) and dl-Cu-Cys (+NIR) groups, the tumor weight decreased over time and reached 110 mg and 5 mg, respectively. In addition, photographs of the tumors removed from the mice were collected at the end of each treatment, further confirming the efficacy of dl-Cu-Cys (+NIR) in inhibiting the growth of tumors (Fig. 7C). The relative tumor volumes were measured in each group during treatment (Fig. S6). The control group showed faster tumor growth, whereas dl-Cu-Cys (−NIR) and dl-Cu-Cys (+NIR) groups, significantly showed significantly delayed tumor growth. Suggesting that NIR light irradiation amplified the therapeutic effects the treatment. These results confirmed the combination of CDT and PTT in the treatment approach to fight against cancer. No significant changes were observed in the body weight of the tumor-bearing mice during treatment, revealing that the nanostructures had negligible side effects (Fig. S7). Besides, representative mouse photos of the tumor site were also recorded to show the tumor recurrence, further indicating the effectiveness of the treatment by the NIR-responsive NPs (Fig. 7D). As demonstrated by infrared thermal images of tumor-bearing mice, tumors injected with dl-Cu-Cys NPs could be effectively heated under the NIR laser irradiation (1.0 W cm^−2^), with their surface temperatures kept at ∼45–48 °C after 5 min irradiation (Fig. 7E). Such mild PTT at low temperature heat treatment (∼43–49 °C) can reduce unnecessary thermal damage to neighboring tissues.^51^ By comparison, the tumor temperature in the mice injected with the PBS solution only increased to 38.8 °C under the same irradiation conditions.

**Fig. 8:**
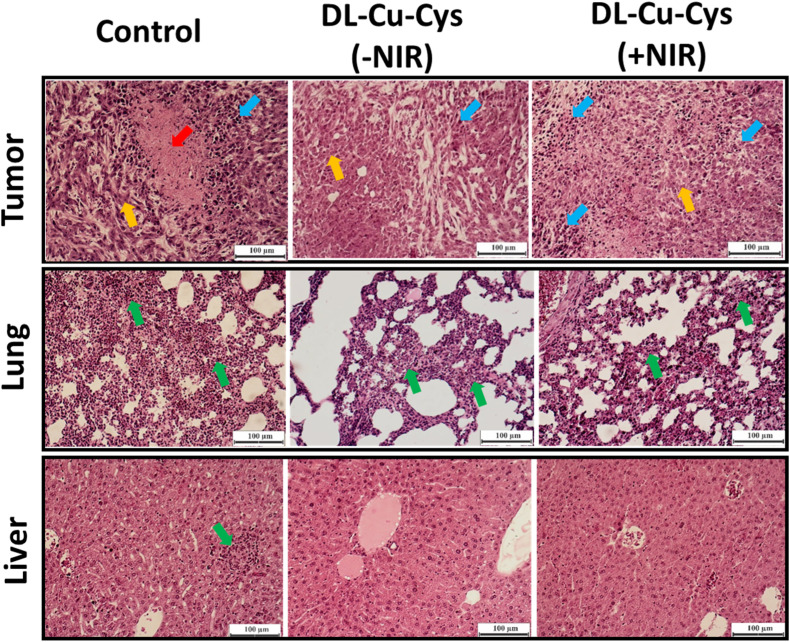
Representative H & E staining of staining images of excised tumor, lung, and liver from different groups at day 14. Scale bar: 100 µm. Pyknosis, apoptosis, and metastatic nodules are shown as blue, yellow, and green arrows respectively. The red arrow shows the shows fibrosis region.

The hematoxylin and eosin staining image (H & E) was used to study tumor treatment by the nanostructures and metastasis to the lungs and liver (Fig. 8). As shown, minimum pyknosis (blue arrows) and apoptosis (yellow arrows) were observed in the dl-Cu-Cys (+NIR) group, confirming the efficiency of the treatment.^52^ In contrast, severe cell proliferation and even a fibrosis region (red arrow) were observed in the control group. The excellent *in vivo* antitumor activity of dl-Cu-Cys (+NIR) encouraged us to further evaluate its potency as anticancer metastasis agent because cancer metastasis is rambunctious for most malignant tumors.^53^ Fewer pulmonary metastatic nodules were observed in dl-Cu-Cys (+NIR)-treated mice than in PBS-treated mice (green arrows). Interestingly, in the case of dl-Cu-Cys (−NIR) lung tissue had severe abnormality and metastatic nodules. No tumor metastasis was observed in mice administered by dl-Cu-Cys (+NIR), where liver metastases were clearly observed in the control group (green arrow). These results indicated that during the *in vivo* process, the dl-Cu-Cys mediated photothermal ablation, effectively elicited systemic immunity response against both tumors recurrence and metastasis.^5,54^

## 4. Conclusion

In this work, the fabrication, characterization, and application of multifunctional dl-Cu-Cys NPs are demonstrated for use as a novel synergistic photothermal and chemodynamic therapy platform against metastatic breast cancer. The dl-Cu-Cys NPs exhibited high photothermal efficiency in increasing localized tumor temperature up to 48 °C and significantly enhancing ROS generation through copper-catalyzed Fenton-like reactions. These two therapeutic mechanisms cooperated in exerting striking biological effects, such as apoptosis and a significant inhibition of cell migration *in vitro*, while *in vivo*, notable results were obtained with a drastic reduction of tumor weight and effective prevention of metastasis to vital organs such as the lungs and liver. These results provide great promise for this nanoplatform in improving therapeutic efficiency while reducing side effects, hence pointing to a path toward the next generation of more effective and patient-friendly cancer treatments. Finally, more efforts toward optimization of clinical translation and long-term safety will further establish dl-Cu-Cys NPs as one the of the most transformative advances against cancer.

## Conflicts of interest

The authors declare no conflict of interest. They have no known competing financial interests or personal relationships that could have appeared to influence the work reported in this paper.

## Supplementary Material

RA-016-D5RA08100A-s001

## Data Availability

All experimental data, including detailed characterization of the nanoparticles, photothermal performance metrics, results of reactive oxygen species (ROS) assays, and full *in vitro* and *in vivo* analysis, are provided in the supplementary information (SI) accompanying this manuscript. Supplementary information is available. See DOI: https://doi.org/10.1039/d5ra08100a.

## References

[cit1] Soerjomataram I., Lortet-Tieulent J., Parkin D. M., Ferlay J., Mathers C., Forman D., Bray F. (2012). Lancet.

[cit2] Jia Y. P., Shi K., Yang F., Liao J. F., Han R. X., Yuan L. P., Hao Y., Pan M., Xiao Y., Qian Z. Y. (2020). Adv. Funct. Mater..

[cit3] Weiss F., Lauffenburger D., Friedl P. (2022). Nat. Rev. Cancer.

[cit4] Shi H., Yan R., Wu L., Sun Y., Liu S., Zhou Z., He J., Ye D. (2018). Acta Biomater..

[cit5] Li H., Wang K., Yang X., Zhou Y., Ping Q., Oupicky D., Sun M. (2017). Acta Biomater..

[cit6] Shanmugam V., Selvakumar S., Yeh C.-S. (2014). Chem. Soc. Rev..

[cit7] Yi X., Yang K., Liang C., Zhong X., Ning P., Song G., Wang D., Ge C., Chen C., Chai Z. (2015). Adv. Funct. Mater..

[cit8] Maleki A., Seyedhamzeh M., Yuan M., Agarwal T., Sharifi I., Mohammadi A., Kelicen-Uğur P., Hamidi M., Malaki M., Al Kheraif A. A. (2023). Small.

[cit9] Tian Q., Xue F., Wang Y., Cheng Y., An L., Yang S., Chen X., Huang G. (2021). Nano Today.

[cit10] Pan Q., Xie L., Liu R., Pu Y., Wu D., Gao W., Luo K., He B. (2022). Int. J. Pharm..

[cit11] Kang J., Jeong H., Jeong M., Kim J., Park S., Jung J., An J. M., Kim D. (2023). J. Am. Chem. Soc..

[cit12] Song W., Zeng J., Ji P., Han Z., Sun Y., Zhang X. (2023). Small.

[cit13] Liu B., Bian Y., Liang S., Yuan M., Dong S., He F., Gai S., Yang P., Cheng Z., Lin J. (2021). ACS Nano.

[cit14] Banci L., Bertini I., Ciofi-Baffoni S., Kozyreva T., Zovo K., Palumaa P. (2010). Nature.

[cit15] Liu J., Yuan Y., Cheng Y., Fu D., Chen Z., Wang Y., Zhang L., Yao C., Shi L., Li M. (2022). J. Am. Chem. Soc..

[cit16] Chan L., Liu Y., Chen M., Su Y., Guo J., Zhu L., Zhan M., Chen T., Lu L. (2023). Adv. Funct. Mater..

[cit17] Guan M., Cheng K., Xie X.-T., Li Y., Ma M.-W., Zhang B., Chen S., Chen W., Liu B., Fan J.-X. (2024). Nat. Commun..

[cit18] Xiong Y., Rao Y., Hu J., Luo Z., Chen C. (2023). Adv. Mater..

[cit19] Bagheri H., Bochani S., Seyedhamzeh M., Shokri Z., Kalantari-Hesari A., Turner R. J., Kharaziha M., Esmaeilzadeh K., Golami M., Zeighami H., Maleki A. (2024). Adv. Ther..

[cit20] Bochani S., Kalantari-Hesari A., Haghi F., Alinezhad V., Bagheri H., Makvandi P., Shahbazi M.-A., Salimi A., Hirata I., Mattoli V. (2022). ACS Appl. Bio Mater..

[cit21] Wang C., Xue H., Zhuang L., Sun H., Zheng H., Wang S., He S., Luo X. (2023). ACS Omega.

[cit22] Muhammad F., Wang A., Miao L., Wang P., Li Q., Liu J., Du J., Zhu G. (2015). Langmuir.

[cit23] Alinezhad V., Ghodsi R., Bagheri H., Beram F. M., Zeighami H., Kalantari-Hesari A., Salarilak L., Mostafavi E., Ahmadian Z., Shahbazi M.-A. (2024). New J. Chem..

[cit24] Liu Y., Ai K., Liu J., Deng M., He Y., Lu L. (2013). Adv. Mater..

[cit25] Cheng Y., Chen Q., Guo Z., Li M., Yang X., Wan G., Chen H., Zhang Q., Wang Y. (2020). ACS Nano.

[cit26] Sun H., Su J., Meng Q., Yin Q., Zhang Z., Yu H., Zhang P., Wang S., Li Y. (2016). Acta Pharmacol. Sin..

[cit27] Bahar E. and Yoon H., in Healthcare, MDPI, 2021, vol. 9, p. 911

[cit28] Tripathy S., Haque S., Londhe S., Das S., Norbert C. C., Chandra Y., Sreedhar B., Patra C. R. (2024). Biomater. Adv..

[cit29] Axet M. R., Philippot K., Chaudret B., Cabié M., Giorgio S., Henry C. R. (2011). Small.

[cit30] Biacchi A. J., Schaak R. E. (2015). ACS Nano.

[cit31] Abdullahi S. S., Güner S., Koseoglu Y., Musa I. M., Adamu B. I., Abdulhamid M. I. (2016). NAMP J..

[cit32] Wu Y., Xu W., Jiao L., Tang Y., Chen Y., Gu W., Zhu C. (2022). Mater. Today.

[cit33] Jana D., He B., Chen Y., Liu J., Zhao Y. (2022). Adv. Mater..

[cit34] Kimber R. L., Lewis E. A., Parmeggiani F., Smith K., Bagshaw H., Starborg T., Joshi N., Figueroa A. I., Van der Laan G., Cibin G. (2018). Small.

[cit35] Li J., Ke W., Wang L., Huang M., Yin W., Zhang P., Chen Q., Ge Z. (2016). J. Control. Release.

[cit36] Yang W., Xia B., Wang L., Ma S., Liang H., Wang D., Huang J. (2021). Mater. Today Sustain..

[cit37] Chudal L., Pandey N. K., Phan J., Johnson O., Lin L., Yu H., Shu Y., Huang Z., Xing M., Liu J. P. (2020). ACS Appl. Bio Mater..

[cit38] Shan J., Yang K., Xiu W., Qiu Q., Dai S., Yuwen L., Weng L., Teng Z., Wang L. (2020). Small.

[cit39] Yuan M., Liang S., Zhou Y., Xiao X., Liu B., Yang C., Ma P., Cheng Z., Lin J. (2021). Nano Lett..

[cit40] Salehi N., Mohammadi A., Alinezhad V., Bochani S., Kalantari-Hesari A., Haghi F., Valdez F. J. S., Buenfil-Chi T. J., Maleki A., Beigi-Boroujeni S. (2025). J. Mater. Chem. B.

[cit41] Azizi R., Kermanian M., Alinezhad V., Kalantari-Hesari A., Yousefiasl S., Maeso L., Orive G., Mohammadi A., Esmaeilzadeh K., Seyedhamzeh M. (2025). J. Mater. Chem. B.

[cit42] Knavel E. M., Brace C. L. (2013). Tech. Vasc. Interv. Radiol..

[cit43] Elmore S. (2007). Toxicol. Pathol..

[cit44] Zhang L., Gao S., Zhang F., Yang K., Ma Q., Zhu L. (2014). ACS Nano.

[cit45] Zhou M., Zhang R., Huang M., Lu W., Song S., Melancon M. P., Tian M., Liang D., Li C. (2010). J. Am. Chem. Soc..

[cit46] Oberley L. W., Oberley T. D. (1988). Mol. Cell. Biochem..

[cit47] Hu R., Fang Y., Huo M., Yao H., Wang C., Chen Y., Wu R. (2019). Biomaterials.

[cit48] Robertson C. A., Evans D. H., Abrahamse H. (2009). J. Photochem. Photobiol. B Biol..

[cit49] Liang C.-C., Park A. Y., Guan J.-L. (2007). Nat. Protoc..

[cit50] Liu K., Zhang L., Lu H., Wen Y., Bi B., Wang G., Jiang Y., Zeng L., Zhao J. (2023). J. Nanobiotechnology.

[cit51] Zhen X., Xie C., Jiang Y., Ai X., Xing B., Pu K. (2018). Nano Lett..

[cit52] Qi Y., Ren S., Ye J., Bi S., Shi L., Fang Y., Wang G., Finfrock Y. Z., Li J., Che Y. (2023). Adv. Healthc. Mater..

[cit53] Zheng J., Ge H., Guo M., Zhang T., Hu Q., Yao Q., Long S., Sun W., Fan J., Du J. (2024). Small.

[cit54] Zhang Y., He X., Zhang Y., Zhao Y., Lu S., Peng Y., Lu L., Hu X., Zhan M. (2021). Chem. Eng. J..

