# Supplementary material for: A novel copper-based nanosystem for augmented breast cancer photothermal and chemodynamic therapy
Source: RSC Adv. 2026 Jan 8;16(3):2167–78. doi: 10.1039/d5ra08100a (PMC12780733; doi:10.1039/d5ra08100a)
Supplement: RA-016-D5RA08100A-s001 [file RA-016-D5RA08100A-s001.pdf]

Supplementary Information (SI) for RSC Advances.
This journal is © The Royal Society of Chemistry 2025

A novel copper-based nanosystem for augmented breast cancer photothermal and chemodynamic therapy

Mohammad Seyedhamzeh[1,#], Farzaneh Falahi[2,#], Ali Kalantari-Hesari[3], Kimia Esmaeilzadeh[1], Laleh Salarilak[1], Hadi Bagheri[1], Shayesteh Bochani[1], Satar Yousefiasl[4], Helia Behrouzfar[5], Mohammad-Esmaeil Akbari[6*], Aziz Maleki[7,1*]

Affiliations:

[1]Zanjan Pharmaceutical Nanotechnology Research Center (ZPNRC), Zanjan University of Medical Sciences, Zanjan 45139-56184, Iran

[2]Department of Medical Biotechnology, School of Medicine, Zanjan University of Medical Science, Zanjan 45139−56111, Iran

[3]Department of Basic Sciences, Faculty of Veterinary Medicine, Bu-Ali Sina University

[4]Department of Pharmaceutical Nanotechnology, School of Pharmacy, Zanjan University of Medical Sciences, Zanjan 45139-56184, Iran

[5]Faculty of Veterinary Medicine, Science and Research Branch of Islamic Azad University, Tehran, Iran

[6]Dental Research Center, Dentistry Research Institute, Tehran University of Medical Sciences, Tehran 1417614411, Iran

[6]Cancer Research Center, Shahid Beheshti University of Medical Sciences, Tehran, Iran.

[7]Department of Pharmaceutical Nanotechnology, School of Pharmacy, Zanjan University of Medical Science, Zanjan 45139−56111, Iran

[#]These authors contributed equally

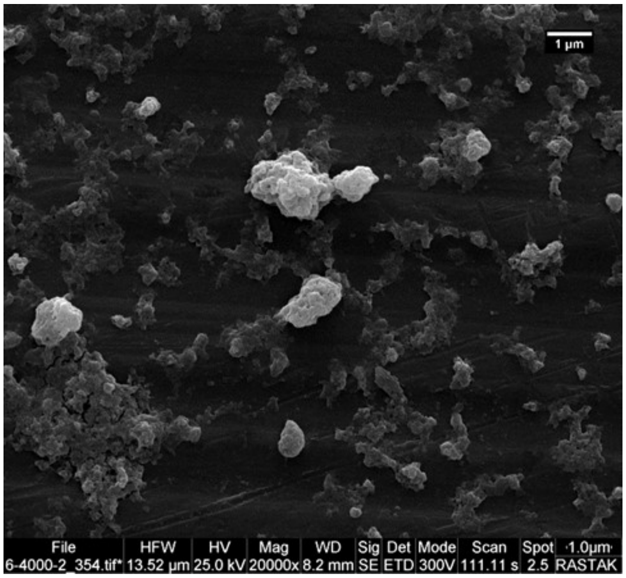

Fig. S1 SEM image of DL-Cu-Cys After incubation with H_2O_2

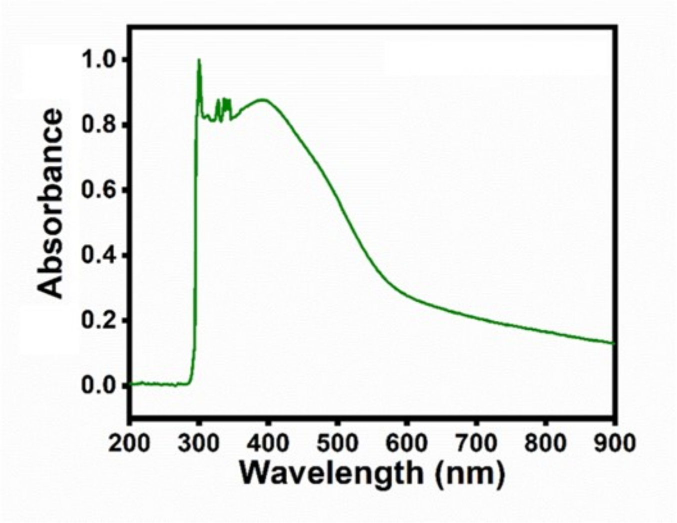

Fig. S2 UV.vis spectrum of DL-Cu-Cys after incubation with H_2O_2

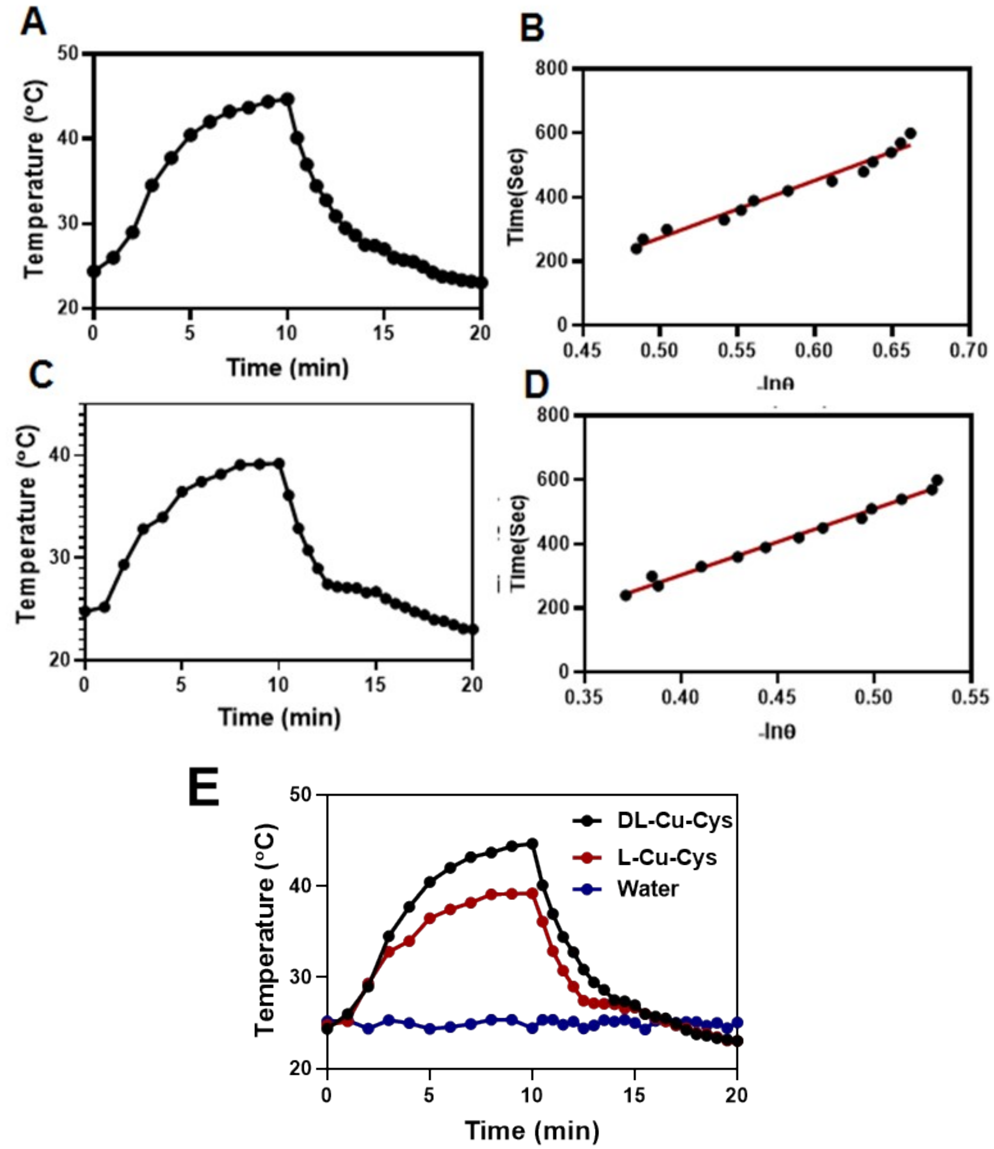

Fig. S3 (A) The photo thermal response of the DL-Cu-Cys with a NIR laser (808 nm, 1.5 w cm^{-2}) for 10 min and the cooling phase of 10 min. **(B)** Linear time data *versus* $-\text{Ln}\,\theta$ measured by the cooling period of Figure 4A and **(C)** The photo thermal response of the L-Cu-Cys with a NIR laser (808 nm, 1.5 w cm^{-2}) for 10 min and the cooling phase of 10 min. **(D)** Linear time data *versus* $-\text{Ln}\,\theta$ measured by the cooling period of Figure 4c (**E**) Temperature curves of DL-Cu-Cys, L-Cu-Cys NPs, and water (808 nm, 1.5 W cm^{-2}).

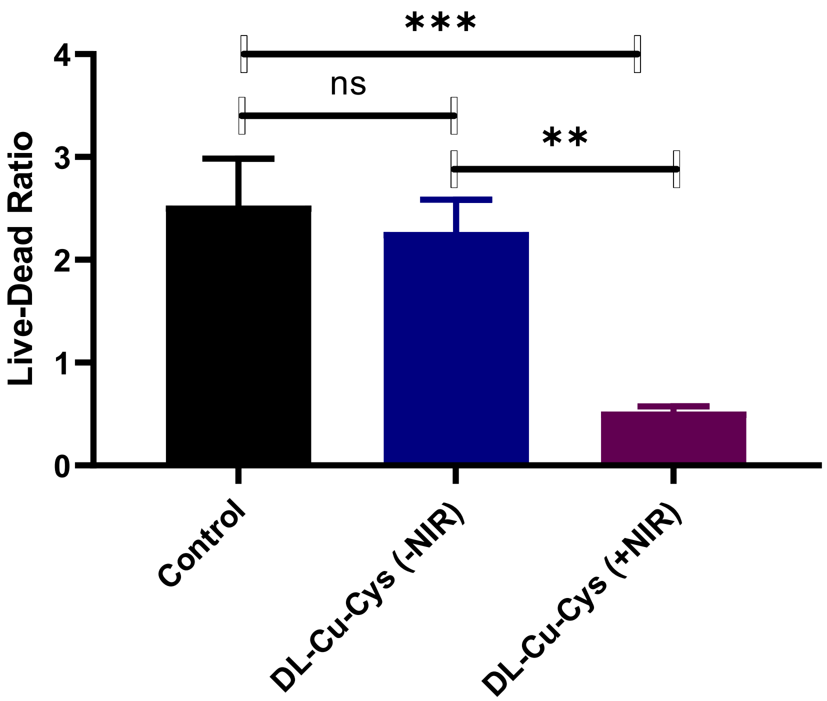

Fig. S4 The live/dead ratio in each groups was quantified. Data are expressed as mean ± SD of three independent replicates. Significant differences are marked with ** $p<0.01$ and *** $p<0.001$.

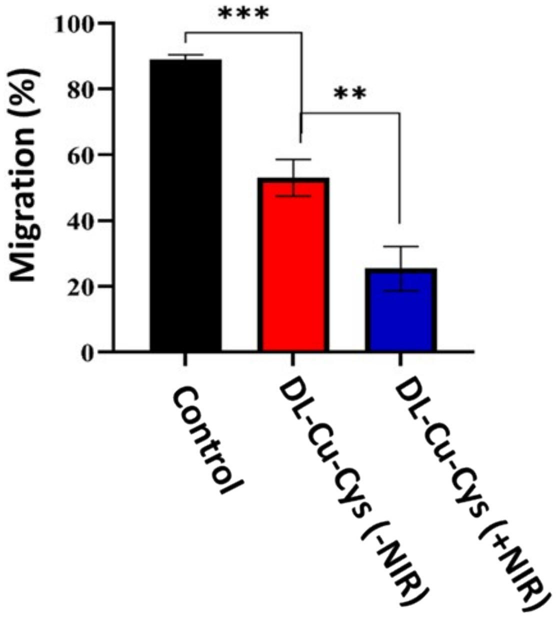

Fig. S5 The percentage of migrated cells in each groups was quantified. Data are expressed as mean ± SD of three independent replicates. Significant differences are marked with ** $p<0.01$ and *** $p<0.001$.

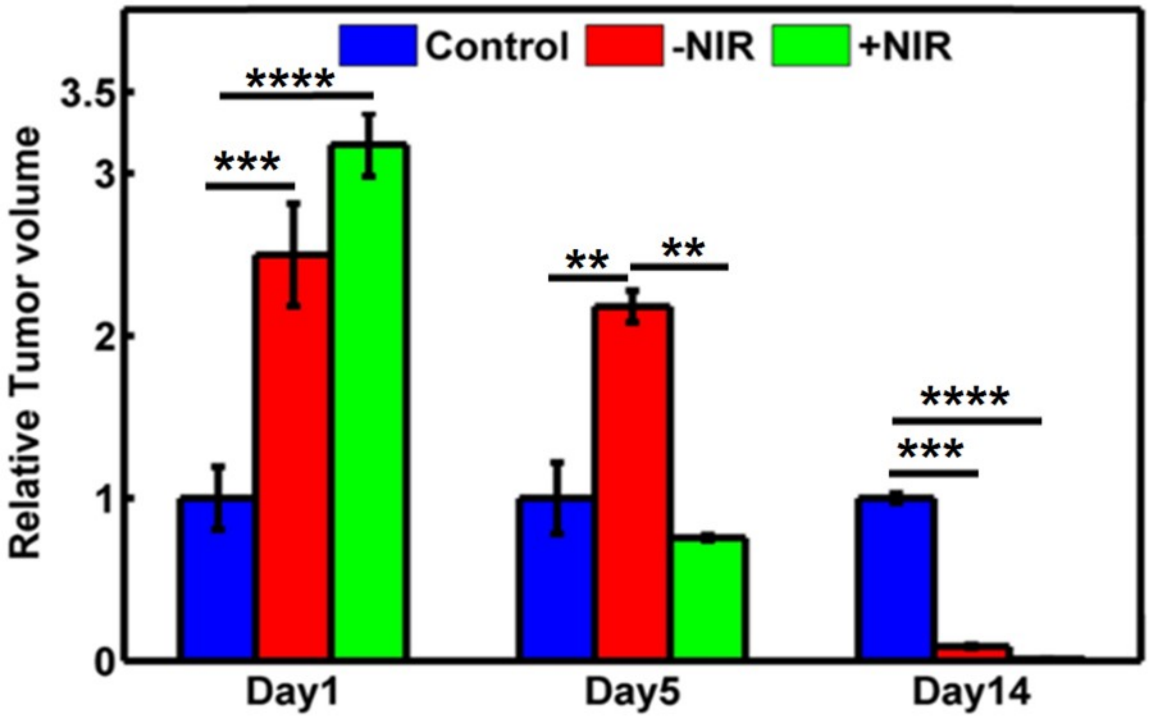

Fig. S6 Monitoring assay for the change of tumor volume (mm^3) over 14 days. Significant differences are marked with ** $p<0.01$ and *** $p<0.001$, and ****$p < 0.0001$.

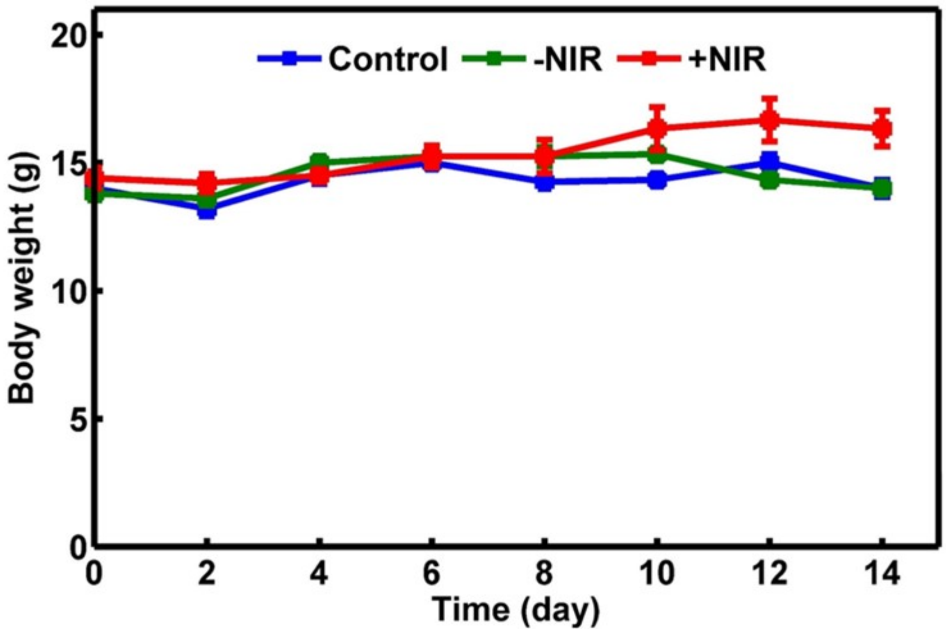

Fig. S7 The measurement of the change of body weight over 14 days**.**
